# Supplementary material for: Characterization of methane microseepage from natural gas reservoirs in mild climate: A case study of Xinchang gas field
Source: PLoS One. 2025 Feb 24;20(2):e0315973. doi: 10.1371/journal.pone.0315973 (PMC11849902; doi:10.1371/journal.pone.0315973)
Supplement: Attachment — Submitted filename: Review of PONE-D-24-03564R1.pdf [file pone.0315973.s003.pdf]

Review of PONE-D-24-03564R1

Accepted with minor revision.

1- I would advise authors to mention some recommendations at the end of both abstract and conclusion based on the results of this study regarding current and future practices in the subjects of the study.

2- Regarding the methodology, I would strongly advise researchers to make and write the chemical and physical properties of soil samples, such as pH, EC, soil texture, preferably in a table. Then, you could discuss how these properties could affect on methane sorption.

3- Also, in the methodology, it is important to mention the period (month and year) in which the samples of study were collected from the field.

Have a good luck.
