# Supplementary material for: Characterization of methane microseepage from natural gas reservoirs in mild climate: A case study of Xinchang gas field
Source: PLoS One. 2025 Feb 24;20(2):e0315973. doi: 10.1371/journal.pone.0315973 (PMC11849902; doi:10.1371/journal.pone.0315973)
Supplement: Attachment — Submitted filename: Comments.docx [file pone.0315973.s001.docx]

This is an interesting research about the characterization of methane microseepage from natural gas reservoirs in mild climate in Xinchang gas field, which contributes as a factor in global climate change. It could be considered for publication after several minor revisions as below:

- The Section **2. Geological 76 Settings of Xinchang Gas Field** should be included in the Section 1. Introduction.
- The Section **3.2. Laboratory Analysis** should be cited some references for the methods used
- Please update the reference to papers published in the last 5 years.
- There are several DOI does not work, please check for any error in the links.
- Some references without DOI, which should be added; and some references were listed with Capital character: Ex. Ref 17, 19…
- The conclusion section should not separate into different paragraphs as in the present manuscript version.
- This phrase should not use in the conclusion section: “The results are consistent with previous studies…”.
