# Supplementary material for: Characterization of methane microseepage from natural gas reservoirs in mild climate: A case study of Xinchang gas field
Source: PLoS One. 2025 Feb 24;20(2):e0315973. doi: 10.1371/journal.pone.0315973 (PMC11849902; doi:10.1371/journal.pone.0315973)
Supplement: Attachment — Submitted filename: Response to Reviews Comments.docx [file pone.0315973.s002.docx]

**Editorial Comments**

1. Please ensure that your manuscript meets PLOS ONE's style requirements, including those for file naming. The PLOS ONE style templates can be found at 
<https://journals.plos.org/plosone/s/file?id=wjVg/PLOSOne_formatting_sample_main_body.pdf> and 
<https://journals.plos.org/plosone/s/file?id=ba62/PLOSOne_formatting_sample_title_authors_affiliations.pdf>

Response: We have edited our manuscript based on the PLOS ONE requirements.

2. In your Methods section, please provide additional information regarding the permits you obtained for the work. Please ensure you have included the full name of the authority that approved the field site access and, if no permits were required, a brief statement explaining why.

Response: No permits were required. The field work was conducted on public lands surrounding the gas wells.

3. Please note that funding information should not appear in any section or other areas of your manuscript. We will only publish funding information present in the Funding Statement section of the online submission form. Please remove any funding-related text from the manuscript.

Response: Removed.

4. Thank you for stating the following financial disclosure: 
   "Natural Science Foundation of China (NSFC Grants No. U2003101, 41872126, and 41373121).
Hangzhou Dianzi University (Grants No. KYS205622065)."

Please state what role the funders took in the study.  If the funders had no role, please state: "The funders had no role in study design, data collection and analysis, decision to publish, or preparation of the manuscript." 
If this statement is not correct you must amend it as needed. 
Please include this amended Role of Funder statement in your cover letter; we will change the online submission form on your behalf.

Response: We have included the amended Role of Funder statement in our cover letter

5. In the online submission form, you indicated that "Data underlying the results presented in the study are available by request for researchers who meet the criteria for access to confidential data."
All PLOS journals now require all data underlying the findings described in their manuscript to be freely available to other researchers, either 1. In a public repository, 2. Within the manuscript itself, or 3. Uploaded as supplementary information.
This policy applies to all data except where public deposition would breach compliance with the protocol approved by your research ethics board. If your data cannot be made publicly available for ethical or legal reasons (e.g., public availability would compromise patient privacy), please explain your reasons on resubmission and your exemption request will be escalated for approval.

Response: We have uploaded the data in supplementary information.

6. When completing the data availability statement of the submission form, you indicated that you will make your data available on acceptance. We strongly recommend all authors decide on a data sharing plan before acceptance, as the process can be lengthy and hold up publication timelines. Please note that, though access restrictions are acceptable now, your entire data will need to be made freely accessible if your manuscript is accepted for publication. This policy applies to all data except where public deposition would breach compliance with the protocol approved by your research ethics board. If you are unable to adhere to our open data policy, please kindly revise your statement to explain your reasoning and we will seek the editor's input on an exemption. Please be assured that, once you have provided your new statement, the assessment of your exemption will not hold up the peer review process.

Response: We have uploaded the data in supplementary information, available to all readers.

7. We note that Figure 1 in your submission contain map/satellite images which may be copyrighted. All PLOS content is published under the Creative Commons Attribution License (CC BY 4.0), which means that the manuscript, images, and Supporting Information files will be freely available online, and any third party is permitted to access, download, copy, distribute, and use these materials in any way, even commercially, with proper attribution. For these reasons, we cannot publish previously copyrighted maps or satellite images created using proprietary data, such as Google software (Google Maps, Street View, and Earth). For more information, see our copyright guidelines: <http://journals.plos.org/plosone/s/licenses-and-copyright>.
 
We require you to either (a) present written permission from the copyright holder to publish these figures specifically under the CC BY 4.0 license, or (b) remove the figures from your submission:
 
a. You may seek permission from the original copyright holder of Figure 1 to publish the content specifically under the CC BY 4.0 license.  
 
We recommend that you contact the original copyright holder with the Content Permission Form (<http://journals.plos.org/plosone/s/file?id=7c09/content-permission-form.pdf>) and the following text:
“I request permission for the open-access journal PLOS ONE to publish XXX under the Creative Commons Attribution License (CCAL) CC BY 4.0 (<http://creativecommons.org/licenses/by/4.0/>). Please be aware that this license allows unrestricted use and distribution, even commercially, by third parties. Please reply and provide explicit written permission to publish XXX under a CC BY license and complete the attached form.”
 
Please upload the completed Content Permission Form or other proof of granted permissions as an "Other" file with your submission.
 
In the figure caption of the copyrighted figure, please include the following text: “Reprinted from [ref] under a CC BY license, with permission from [name of publisher], original copyright [original copyright year].”
 
b. If you are unable to obtain permission from the original copyright holder to publish these figures under the CC BY 4.0 license or if the copyright holder’s requirements are incompatible with the CC BY 4.0 license, please either i) remove the figure or ii) supply a replacement figure that complies with the CC BY 4.0 license. Please check copyright information on all replacement figures and update the figure caption with source information. If applicable, please specify in the figure caption text when a figure is similar but not identical to the original image and is therefore for illustrative purposes only.
The following resources for replacing copyrighted map figures may be helpful:
 
USGS National Map Viewer (public domain): <http://viewer.nationalmap.gov/viewer/>
The Gateway to Astronaut Photography of Earth (public domain): <http://eol.jsc.nasa.gov/sseop/clickmap/>
Maps at the CIA (public domain): <https://www.cia.gov/library/publications/the-world-factbook/index.html> and <https://www.cia.gov/library/publications/cia-maps-publications/index.html>
NASA Earth Observatory (public domain): <http://earthobservatory.nasa.gov/>
Landsat: <http://landsat.visibleearth.nasa.gov/>
USGS EROS (Earth Resources Observatory and Science (EROS) Center) (public domain): [http://eros.usgs.gov/#](http://eros.usgs.gov/)
Natural Earth (public domain): <http://www.naturalearthdata.com/>

Response: We have redrawn the figure to avoid any potential copyright issue.

**Reviewers’ Comments**

Reviewer #1: This document provides additional comments and considerations for the manuscript titled "Characterization of methane microseepage from natural gas reservoirs in mild climate: a case study of Xinchang gas field." The comments focus on potential concerns regarding duplicate publication, research ethics, and publication ethics.
Duplicate Publication: There is no indication that the results presented have been published elsewhere, which is positive. However, the authors should explicitly clarify if any part of the work, including data, figures, or substantial portions of the text, has been or is under consideration for publication in another venue. This clarification is essential to avoid issues of self-plagiarism and duplicate publication.

Response: We hereby clarify that no part of this work, including its data, figures, and texts in length, has been published or under consideration for publication in another venue.

Research Ethics: The methodology section provides detailed information on data collection and analysis. Nonetheless, it would enhance the manuscript's integrity if the authors included a statement on ethical considerations, especially regarding environmental impact assessments of the study area. Given the environmental sensitivity of methane emissions, outlining measures taken to minimize potential harm or disturbance during the study would be beneficial.

Response: This field work involved in this manuscript include methane flux monitoring and sampling in shallow earth (1.5m). These activities do not cause additional methane emissions. Disturbance on local flora, fauna, habitat, and water bodies are minimal. Therefore, we are confident to state that environmental impacts on the study area are negligible.

To our knowledge, it doesn’t seem customary for such statement to appear in the manuscript. If it needs to be, please let us know.

Publication Ethics: The manuscript should adhere to high standards of publication ethics, including full transparency about the contributions of each author and the disclosure of any potential conflicts of interest. While the manuscript includes funding information, a more detailed statement regarding the authors' adherence to ethical guidelines in conducting and reporting their research is recommended.
In conclusion, while the study presents valuable insights into methane microseepage from natural gas reservoirs in a mild climate, addressing the above concerns can significantly strengthen the manuscript. Ensuring clarity on these aspects would enhance the manuscript's contribution to the field and its ethical and scientific rigor.

Response: Our statement to dual submission and plagiarism has been provided in the first response. This work does not involve any human or animal research, so there is nothing to declare on this aspect. We herby declare, again, that this work adheres to all ethical guidelines of PLOS.

And again, to our knowledge, it doesn’t seem customary for such statement to appear in the manuscript. If it needs to be, please let us know.

The study on "Characterization of methane microseepage from natural gas reservoirs in mild climate: a case study of Xinchang gas field" provides significant insights into methane emissions and their implications for climate change. The comprehensive analysis of methane flux, hydrocarbon composition, and carbon isotope profiles in the Xinchang gas field is commendable for its depth and detail. Here are some additional comments and suggestions for further strengthening your manuscript:
Integration with Global Data: While your study provides critical data on methane microseepage in the Xinchang gas field, integrating these findings with global datasets on methane emissions could offer a broader perspective on the significance of your results. Comparing your data with methane flux measurements from different geological settings worldwide could contextualize the unique aspects of methane microseepage in mild climates.

Response: We have elaborated on the comparison of this study with global dataset in Section 3.4.

Methodological Advancements: Your methodology, combining field measurements and laboratory analysis, is robust. However, discussing any potential limitations of your techniques and how they compare with other contemporary methods could enhance the reliability of your findings. For instance, detailing the calibration process of the greenhouse gas analyzer and static gas collection chambers could provide clarity on the accuracy of your measurements.

Response: The calibration of the GHG analyzer is against the background concentrations in air.

Microbial Activity Analysis: Given the significant role of microbial activities in methane consumption, a more detailed analysis of the microbial communities present in the soil samples could provide insights into the biodegradation processes affecting methane flux. This could involve genomic or metagenomic analyses to identify methanotrophic communities and their functional capabilities.

Response: We agree with this comment that microbial activity is critical to methane consumption. Our research has found out confirm negative background methane flux, suggesting in warm climate where microbial communities are active, the soil acts as a sink of methane. Genomic or metagenomic analyses will certainly provide insights in the functionality of the microbial community in Xinchang gas area, but this is already beyond the scope of our project and expertise in the field of chemistry and environmental science. To the purpose of this project, identifying the ensemble effect of the microbial community on methane flux should suffice. We will leave the follow-up to future research and apply for new fundings for it to happen.

Impact of Climate Change: Considering the implications of methane on climate change, an analysis of how future climate scenarios might affect methane microseepage patterns in the Xinchang gas field and similar environments would be valuable. This could involve modeling studies to predict changes in methane flux under various climate change projections.

Response: We certainly agree that climate change and GHG sources can have mutual impact on each other. One example is warm weather and mountain pine beetle infestation – warm weather increases the survival rate of the beetle community in winter, which in turn cause more severe destruction of pines and wildfires out of dead, dry trees.

We added some discussion on the potential impact of climate on methane microseepage, most notably on microbial activity and its role as methane sink, in the end of Section 3.4. However, analysis on a detailed fashion is beyond our expertise, and its scope is big enough for many more publications.

Policy and Management Implications: Expanding the discussion on the implications of your findings for methane management and climate change mitigation policies could make your study more impactful. Suggestions for policy interventions or management strategies to mitigate methane emissions from natural gas reservoirs could provide practical value to your research.

Response: Policy implications have been added to the new Section 3.5.

Longitudinal Studies: Mentioning the potential for longitudinal studies to observe changes in methane microseepage over time, especially in the context of ongoing climate change and human interventions in natural gas fields, could highlight the importance of continued research in this area.

Response: We agree with this comment and are more than happy to follow up on observation over time. To our knowledge, seasonable variation clearly exists for methane microseepage. Monitoring methane emissions over a long period of time are also critical to understanding the evolving climate forcing and continuous methane management.

However, as the project that supports this work has concluded, we will have to apply for new fundings for the follow-up to happen.

These comments are intended to complement the strengths of your manuscript and suggest avenues for further enhancement. Your research contributes significantly to our understanding of methane emissions from natural gas reservoirs and their climate implications, highlighting the need for comprehensive monitoring and management strategies.

Response: Thank you very much.

Reviewer #2: Please double check the manuscript and revise it carefull before re-submit to the Journal:
- The Section 2. Geological 76 Settings of Xinchang Gas Field should be included in the Section 1. Introduction.

Response: Section 2 has merged into Section 1.

- The Section 3.2. Laboratory Analysis should be cited some references for the methods used

Response: Methods used for flux monitoring and lab analysis have been properly cited.

- Please update the reference to papers published in the last 5 years.

Response: In the field of methane microseepage, there aren’t that many relevant papers in the most recent years. We have added as many relevant ones as we can.

- There are several DOI does not work, please check for any error in the links.

Response: We have corrected errors in DOI links.

- Some references without DOI, which should be added; and some references were listed with Capital character: Ex. Ref 17, 19…

Response: We have provided as many DOI as we can and fixed the capital letters. However, some journal papers (in Chinese) don’t have DOI.

- The conclusion section should not separate into different paragraphs as in the present manuscript version.

Response: Revised.

- This phrase should not use in the conclusion section: “The results are consistent with previous studies…”.

Response: This phrase has been edited.
