# Supplementary material for: Characterization of methane microseepage from natural gas reservoirs in mild climate: A case study of Xinchang gas field
Source: PLoS One. 2025 Feb 24;20(2):e0315973. doi: 10.1371/journal.pone.0315973 (PMC11849902; doi:10.1371/journal.pone.0315973)
Supplement: Attachment — Submitted filename: response to comments revision 3.docx [file pone.0315973.s005.docx]

Reviewer #1: The author's response to the feedback provided in the initial review demonstrates a commendable effort to address the reviewers' concerns and significantly improve the manuscript. The revisions made, particularly in the methodological section and the integration of additional references, have enhanced the scientific rigor of the study. The addition of a detailed comparison with global datasets on methane emissions strengthens the contextualization of the study's findings, allowing for a broader application of the results. Furthermore, the clarification regarding ethical considerations and environmental impacts contributes to the transparency and credibility of the research. The response to the suggestions on the microbial activity analysis and the potential impacts of climate change reflects a thoughtful approach to acknowledging the study's limitations while outlining future research directions. The restructuring of certain sections and the refinement of the conclusion have improved the manuscript's overall coherence and clarity. In summary, the authors have successfully addressed the critical points raised, resulting in a more robust and scientifically sound manuscript that makes a valuable contribution to the field.

Response: Thanks very much for the comment.

Reviewer #3: I would advise authors to mention some recommendations at the end of both abstract and conclusion based on the results of this study regarding current and future practices in the subjects of this study.

Response: A few recommendations have been added to both abstract and conclusion.
