# Supplementary material for: Characterization of methane microseepage from natural gas reservoirs in mild climate: A case study of Xinchang gas field
Source: PLoS One. 2025 Feb 24;20(2):e0315973. doi: 10.1371/journal.pone.0315973 (PMC11849902; doi:10.1371/journal.pone.0315973)
Supplement: Attachment — Submitted filename: Response to Reviewers 4.docx [file pone.0315973.s006.docx]

Reviewer #1: The study on methane microseepage from the Xinchang gas field provides valuable insights into geological methane emissions in mild climates. The methodology, including soil sampling and methane flux monitoring, is thorough and appropriate for identifying geochemical anomalies and migration patterns. However, the paper could improve its discussion on the limitations of the study, particularly regarding the impact of local climate variability on methane flux. The absence of long-term monitoring limits the understanding of temporal variations in microseepage. Additionally, the discussion could further explore the role of microbial activity in methane oxidation, which may significantly influence surface flux measurements. Furthermore, while the use of static gas collection chambers is common, complementing these measurements with remote sensing data could enhance spatial coverage and detection sensitivity. The study's findings align with existing literature, yet it would be beneficial to compare the results with regions having different climatic conditions to provide a more comprehensive understanding of methane microseepage behavior.

Response:

Thanks for the constructive comments.

The authors completely agree that long-term monitoring could provide more insights on the evolvement of methane microseepage over time. However, this project has already been ended, so the follow-up monitoring could only be conducted once new fundings can be secured. Meanwhile, there are other studies that focused on seasonal variations in methane microseepage, forming reasonable theories that are also applicable to this case. Therefore, the authors have now developed more discussions on potential seasonal variations in methane microseepage in Xinchang.

Regarding microbial activity in methane oxidation, it certainly has significant impact on methane flux. In this work, the authors have taken it into account, by measuring background methane flux outside the test zones. This background flux is mainly due to the ensemble effect of microbe living in soil. Meanwhile, the authors have also conducted tests to rule out the possibility of notable biogenic methane in the soil. In this way, the authors feel it is safe to draw a high-level conclusion on the role of microbial activity on methane flux, and on the existence and extent of thermogenic methane microseepage from the gas reservoir. Naturally, developing an understanding on the exact methane-related behavior of soil microorganisms are crucial to methane management, yet it is outside the scope of this work and beyond the engineering expertise of the authors. The authors have pointed this topic out, as potential future work for interested microbiologists.

Lastly, the authors agree that complementing in-situ monitoring with remote sensing could enhance the effectiveness of methane monitoring. Yet again, remote sensing would be a whole new project to this one, so the authors provide some brief discussion on it. The authors have also provide comparison of this work with previous ones on different geological and climatic conditions.

Overall, the authors completely agree with the constructively comments provided by the reviewer. Addressing these limitations would certainly improve the general effectiveness in methane flux monitoring, but the work will be beyond the scope of this work and require multidisciplinary expertise. Therefore, the authors have made every effort possible to provide detailed discussions on these limitations of this study and shed light on potential future work.

Questions:
1. How might seasonal variations in temperature and precipitation affect methane microseepage in the Xinchang gas field?

Response: As suggested by a series of studies, such as ref [10] and [11] cited in this manuscript, temperature and precipitation may affect methane microseepage flux in two competing ways. On one hand, cold temperature and arid weather tend to hamper microbial methanotrophic consumptions, which increases net methane flux, as observed in [11]. On the other hand, extreme cold weather may lead to snow covers thick enough to block methane flux, as observed in [10]. These two factors are competing with each other, leading to dynamic seasonal variation patterns in different regions.

As stated in Section 1, Xinchang field is located in a mild climate that never sees daily average temperature below 0 throughout the year. It has a relatively cold and dry winter, which leads to lower microbial activities and thus higher methane flux, yet is never cold enough to allow for the presence of snow cover that blocks methane from entering the atmosphere. Therefore, it is suggested that Xinchang field will see higher methane flux in the winter.

2. What are the potential biases introduced by short-term methane flux measurements, and how can they be mitigated?

Response: As revealed in this work and many others, short-term methane flux can manifest in various patterns. This can be explained by complicated geological micro-structures. As a result, short-term methane flux may come with a great extent of uncertainty, which may be mitigated in two ways.

Firstly, long-term monitoring over a large area may be executed. This would lead to more accurate and thorough monitoring, but is naturally expensive and labor-intensive. Secondly, in-situ monitoring may be coupled with remote sensing techniques, which leads to the third comment point of the reviewer.

Thus, the authors have expanded the section 3.5 in the manuscript regarding this and the next comment of the reviewer.

3. How could the integration of remote sensing techniques improve the detection of methane microseepage compared to traditional methods?

Response: Remote sensing can be coupled with traditional in-situ monitoring to improve the accuracy of methane microseepage surveillance. Traditional in-situ monitoring over a large region can be labor-intensive and expensive. In this sense, remote sensing can effectively provide an overview of atmospheric methane concentration on national or regional scale, pinpointing where atmospheric methane anomaly occurs. With the guidance of remote sensing results, in-situ monitoring can now be used to identify exact methane microseepage hotspots in an area with known abnormal atmospheric methane concentration distribution.

4. In what ways does microbial activity in soil influence the measurement of methane flux, and how was this accounted for in the study?
Response: As stated in Section 3.3, both methane coefficient (C1/C2+) and Carbon isotope ratio, analyzed in this work, can help to identify the origin of methane and other hydrocarbons. The results of both indicators suggest that the methane and hydrocarbons sampled from our test sites are thermogenic, instead of biogenic. That is to say, this hydrocarbon mix, methane included, originates from underground fossil fuel reservoir, rather than from living microorganisms in soil.

On the other hand, the background methane flux recorded outside the gas field zone in our work is negative. This is clearly a sign of methane absorption from atmosphere to soil, by no other means than microbial activities.

Naturally, to determine the methane microseepage flux, that is the methane migrated from reservoir to atmosphere, the background methane flux is deducted from the flux measured on test sites.

5. How do the results from the Xinchang gas field compare to other regions with different geological or climatic conditions?

Response: The entire Section 3.4 discusses comparison of this study with previous ones. We have reviewed ten studies on this topic and compared the results with Xinchang field. In short, the net methane flux from Xinchang gas field is considered very weak, compared to other oil and gas reservoirs. The mild and humid climate of Xinchang tends to facilitate microbial activities, increasing soil methane absorption.
